# Slaughter of pregnant goats for meat at Nsukka slaughterhouse and its economic implications: A public health concern

**DOI:** 10.14202/vetworld.2018.1139-1144

**Published:** 2018-08-17

**Authors:** Onyinye Josephine Okorie-Kanu, Ekene Vivienne Ezenduka, Christian Onwuchokwe Okorie-Kanu, Chidiebere Ohazurike Anyaoha, Chukwuebuka Anselm Attah, Toochukwu Eleazar Ejiofor, S. Onyinye Onwumere-Idoloh

**Affiliations:** 1 Department of Veterinary Public Health and Preventive Medicine, University of Nigeria, Nsukka, Nigeria; 2 Department of Pathology, Michael Okpara College of Agriculture, Umudike, Nigeria; 3 Department of Agric, Education, University of Nigeria, Nsukka, Nigeria; 4 Department of Agricultural Technology, School of Agriculture, Delta State Polytechnic, Ozoro, Delta State, Nigeria

**Keywords:** economic implications, fetuses, low meat quality, pregnant goats, public health, unwholesome

## Abstract

**Aim:**

This study was conducted to determine the incidence rate of the slaughter of pregnant goats in Nsukka slaughterhouse, which has become a major cruel occurrence in Nigeria, as well as it’s economic and public health implications.

**Materials and Methods:**

All the goats slaughtered at Nsukka slaughterhouse over a period of 3 months (February-April, 2017) were screened. The data collected were: total number of goats slaughtered, age, breed and sex of goats slaughtered, pregnancy status of the goats, and sex of the fetuses observed, and gestational age of the fetuses estimated by crown-rump length.

**Results:**

In the 3-month study, a total of 684 goats were slaughtered, of which 617 (90.2%) were females. 364 (59%) of the females slaughtered were pregnant, and more than 80% of the gestations were in the second and third trimesters. Of 661 fetuses recorded, 320 (48.3%) were males, and 341 (51.7%) were females with 438 (66.3%) predominantly twins. At the cost of ₦ 6,000 ($16) and ₦ 8,000 ($20) for male and female kids, respectively, a total of ₦ 4,648,000 ($11,620) was lost in just one slaughterhouse in 3 months.

**Conclusion:**

This study shows that there is a high rate of slaughter of pregnant goats in Nsukka slaughterhouse with a tremendous economic loss, and most chevon sold in Nsukka are unwholesome and of low meat quality.

## Introduction

Nigeria occupies 923,768 km^2^ located on the Gulf of Guinea in West Africa. With the fall in the price of oil in the Global market and a negative effect on the economy of Nigeria that relies mainly on oil, the resultant effect of recession, loss of jobs and unemployment, has made over 70% of the population to be engaged in agriculture [1]. Growth in the livestock sector has consistently exceeded that of the crop sector [2]. The total demand for animal products in developing countries is expected to be more than double by 2030 [2], yet livestock production in Nigeria is still at its primordial stage. Animals are kept with little or no veterinary attention and majority suffer from different infectious diseases such as contagious bovine pleuropneumonia (CBPP), foot and mouth disease (FMD) of cattle, peste des petits ruminants (PPR) of goats and trypanosomosis [3]. This is one of the obstacles to the development of the livestock industry in Nigeria [3].

Although livestock production in Nigeria is still at the subsistence level, it provides all the locally produced milk and over 70% of the national meat supply, as well as serves as a source of financial security/income as well as food, farm energy, manure, and transport to the small-holding farmer [4]. Apart from its source of employment, occasionally livestock are used for rituals, religious festivals, and ceremonies. Livestock production contributes 6-8% of the GDP and accounts for one-third (20-25%) of Nigeria’s agricultural GDP [5].

Livestock farming in Nigeria is faced with many other challenges. Among the challenges are inadequate quantity and low quality of available feed, inadequate water supply and uneven water distribution, unstable supply of external inputs including veterinary supplies and concentrates, low genetic potential of native livestock with regard to feed conversion and reproduction, and lack of concrete national policies that would give focus and direction to livestock production and research [6]. All these, force livestock production to continue to thrive at a subsistence level.

In Nigeria, there is more preference for goat production than the other ruminants. Sokoto red breed of goats (indigenous breed in Nigeria, scientific name is not known) is the predominant breed kept in Nigeria [7]. Although they have a twinning ability, they are of poor genetic potential (as evidenced by their relatively small size) and are kept under poor resource investment and raised subsistently. As a result of lack of breeding programs and other veterinary services, there is no record on the breeding pattern of these animals. They are kept to roam and graze in the fields. They interbreed, and no effort is made to separate the pregnant ones. For financial reasons, these farmers who keep them as an asset sell the robust looking ones for good price (to livestock traders and butchers), without consideration to their pregnancy status.

In most developed countries such as the USA, Australia, and UK, the annual meat consumption rate is 120.2, 111.5, and 84.2 kg/person/year, respectively, while in developing countries of mainly Africa such as Nigeria, Sierra-Leone, Rwanda, and Burundi, it is 8.8, 7.3, 6.5, and 5.2 kg/person/year, respectively. This is, however, lower than the world average meat consumption rate of 41.9 kg/person/year [8]. FAO estimated that, in 2030, the average annual meat consumption in developing countries including Nigeria is projected to increase to 37 kg per person per year [2]. Nigerian population was estimated at over 174 million in 2013 [4] while cattle, sheep, and goat populations were recently estimated at 19.2 million, 38.5 million, and 57.4 million, respectively [2], The human population in Nigeria is growing with an estimated increment of 3.5% per year while livestock resources grow between 0.8 and 2.9% per year [9].

At present, the low livestock growth rate in Nigeria compared to the human growth rate is putting pressure on animal protein demand. This consequently leads to the slaughter of prime breeding animals including pregnant animals for meat [10]. This is an evitable human-made constraint to the development of the livestock industry in Nigeria.

The practice of slaughtering pregnant animals is seen routinely in almost all abattoirs and slaughterhouses in Nigeria, and the ugly effect of foetal losses resulting from this barbaric act has been reported among large (cattle) and small (sheep and goats) ruminants from several abattoirs and slaughterhouses across Nigeria’s senatorial zones – South South [9], South West [11], South East [12], North Central [13-17], North West [18], and North East [10,19]. Apart from the resultant wastage of scarce protein available to consumers and a decrease in the livestock growth capacity of the country due to low herd replacement rates, it depicts the highest level of cruelty to animals. Moreso and importantly, the quality of meat resulting from this condemnable act is low and unwholesome. Unfortunately, the consumers of meat in Nigeria as well as many other developing countries are not aware of the source or quality of meat being sold to them for consumption unlike in the developed countries where increasing public interest in sustainable, high-quality, and safe food can be observed and where consumers inspect food production processes taking into account aspects such as animal welfare and other social and ethical attributes [20].

The associated economic losses from pregnancy wastage are enormous and have been reported by many researchers in Nigeria [9,10,13,14,17,19]. These losses constrain the active contribution of livestock to the gross domestic product in the country.

Fetuses from pregnant goats slaughtered for meat are discovered every day at Nsukka slaughterhouse, Enugu State. Lack of implementation of the Meat Inspection Act of 1968 that prohibits the slaughter of pregnant animals for meat in Nigeria is attributed to the cause of this menace. There is a need to investigate the extent of fetal wastage resulting from the slaughter of pregnant goats for meat at Nsukka slaughterhouse, Enugu State, Nigeria. This study was conducted to determine the incidence rate of the slaughter of pregnant goats in Nsukka slaughterhouse, which has become a major cruel occurrence in Nigeria, as well as it’s economic and public health implications.

## Materials and Methods

### Ethical approval

The study was conducted in accordance with the Ethics and Regulation Guiding the Use of Animals as approved by the University of Nigeria, Nsukka.

### Study area

The research work was carried out at Nsukka slaughterhouse, Enugu State, Nigeria. It lies on the geographical coordinates of 6° 51’ 24’’ N (latitude) and 7° 23’ 45’’ E (longitude). It is the site of the University of Nigeria, the first indigenous University in Nigeria. The presence of the University since 1960 has attracted a huge number of people to the area so that the population of Nsukka as of 2006 is 309,633 [21]. Meat (cattle, pig, goat, and chicken) consumption is part of the food habit/culture of the population. Nsukka slaughterhouse has the largest slaughter capacity of animals in Enugu State, and all the meat consumed in Nsukka are processed there.

### Study population, design, and duration

The study population comprised all the goats slaughtered at Nsukka abattoir. It included the two most common breeds of goat found in Nigeria; the Sahelian goats or the Sokoto red goats and the West African Dwarf (WAD) goats. The Sokoto red goats come from Northern Nigeria while the WAD is particularly reared by the local breeders in the Southern region of Nigeria.

A cross-sectional study design which lasted for 3 months (February-April, 2017) was used for this study.

### Data collection

Data were collected from the abattoir daily from 6 am to 9 am for a period of 3 months except on Sundays and Mondays when the butchers go to source for goats from the North of Nigeria. Information on the number, age, breed, sex, and pregnancy status of the goats slaughtered was collected and recorded. The number, sex, and gestational age of the fetuses were also collected and recorded. The gestational ages of the fetuses were estimated by the crown-rump length using the formula provided by Singh *et al*. [22].

Y=24.42+0.39x

Where,

Y=Gestational age of the fetus.

X=Crown-rump length in millimeters.

The estimated gestational ages of the fetuses were used to allot the fetuses into appropriate trimesters depicting the three stages of gestation.

Economic losses from the goat fetal wastage were analyzed at an estimated cost of 8,000 nairas ($20) for female kid at birth and 6,000 nairas ($15) for male kid at birth in the study area (Oral communication with the butchers and goat traders).

### Statistical analysis

Data generated were analyzed using descriptive analysis such as simple averages and percentages.

## Results

In the period under study, a total of 684 goats were slaughtered at Nsukka slaughterhouse. A total of 60, representing 8.8% were of the WAD breed while Sokoto red breed accounted for 624, representing 91.2% of the total slaughter. Proportionately, more females (90.2%) than males (9.8%) were slaughtered, and it was consistent across the 3 months of study (Table-1).

**Table-1:** Breed and sex of goats slaughtered.

Month	Goats slaughtered		Breed				Sex			
		
	Number	Proportion	Sokoto red	Proportion	West African Dwarf	Proportion	Female	Proportion	Male	Proportion
February	208	30.4	193	92.8	15	7.2	187	89.9	21	10.1
March	211	30.8	191	90.5	20	9.4	192	91.0	19	9.0
April	265	38.7	240	90.5	25	9.4	238	89.8	27	10.2
Total	687		624	91.2	60	8.8	617	90.2	67	9.8

The age distribution of the females slaughtered showed that a total of 103 (16.7%), 126 (20.4%), 197 (31.9%), 163 (26.4%), and 28 (4.5%) were within the age ranges (in years) of 0-<1, 1-<2, 2-<3, 3-<4, and >4, respectively (Table-2).

**Table-2:** Age distribution of female goats slaughtered at Nsukka slaughterhouse.

Age distribution (years)	Number	Proportion (%)
0-<1	114	16.7
1-<2	140	20.5
2-<3	218	31.9
3-<4	181	26.5
>4	31	4.5
Total	684	

A total of 364 pregnant does were slaughtered, representing 59% of the total (617) females, and it was consistently higher across the 3 months of study when compared to the non-pregnant does (Table-3). When the pregnancy status of these female goats was analyzed using the different stages of pregnancy, 66 were in their 1^st^ trimester (0-50 days) representing 18.1% of the pregnant population, 189 were in their 2^nd^ trimester (50-100 days), and 109 in their 3^rd^ trimester (100-150 days) representing 51.9% and 29.9%, respectively (Table-4).

**Table-3:** Female goats slaughtered with the type and sex of fetuses wasted.

Month	Female goats slaughtered		Fetal types				Sex of fetuses wasted		
		
	No. pregnant	No. not pregnant	Single	Twin	Triplet	Quadruplet	Female	Male	Number
February	127	60	51	70	5	1	108	102	210
March	113	79	36	66	10	1	113	89	202
April	124	114	21	83	19	1	120	128	248
Total (%)	364 (59)	253 (41)	108 (16.4)	219 (66.3)	34 (15.4)	3 (1.8)	341 (51.6)	319 (48.3)	660

**Table-4:** Pregnancy status of the female goats slaughtered.

Trimesters	Pregnant does	Proportion (%)
1^st^ trimester	66	18.1
2^nd^ trimester	189	51.9
3^rd^ trimester	109	29.9
Total	364	

The total number of fetuses wasted at Nsukka slaughterhouse within the 3-month study period was 660, with 210 (31.8%), 202 (30.6%), and 248 (37.5%) discovered in February, March, and April, respectively (Table-3). The fetuses (438) were predominantly twins (66.3%) while 16.4%, 15.5%, and 1.8% were single, triplet, and quadruplet fetuses, respectively (Table-3).

The estimated economic loss from 660 fetuses (comprising 319 [48.3%] males and 341 [51.7%] females) wasted in Nsukka slaughterhouse for just 3 months is about 2,728,000 naira ($6,820) for the female while 1,920,000 naira ($4,800) for the male, making a total of 4,648,000 naira ($11,620).

## Discussion

From the study, the breed distribution showed the slaughter of more Sokoto red at Nsukka slaughterhouse than the West African breed of goats. This could be attributed to the fact that Nsukka town where the slaughterhouse is located is mainly inhabited by civil servants whose animal protein demand (by virtue of a relative increase in wealth and a higher standard of living) is higher than what the available WAD goats kept by the rural households can meet. Therefore, livestock traders and butchers to meet their high protein demand, procure Sokoto red goats from the Northern region of Nigeria where over 70% of the small ruminants in the country (which are sheep and mainly Sokoto red goats) are reared [6].

Our study revealed that 90% of the goats slaughtered are females. This is worrisome because, ideally, sound economic livestock management demands that animals sold for slaughter should be mainly males and reproductively inactive females [23]. One would expect that a country with its diet consisting mainly of very low protein and high carbohydrate levels [24], with annual meat consumption rate of 8.8 kg/person/year [7] and high protein-energy malnutrition record [25] would put in place all measures to increase the national herd size by safeguarding its reproductive female stock. However, high rates of slaughter of female livestock ranging from 42 to 84% have been recorded by several researchers across Nigeria [10,14-17]. More importantly, 79% of the females slaughtered were actively reproductive does as they were found to be between the ages of 1 and 4 years old. This corroborates the assertions made by some authors [12,13] that fetuses are lost due to the slaughter of reproductively active dams in both large and small ruminants in Nigeria.

The female to male birth ratio as observed in this study is 1.07:1. The main reason for the slaughter of more female goats generally than males in the study area, even when there is no significant difference in the ratio of female to male births calls for serious concern.

The study further revealed that the proportion of slaughter of pregnant female goats to the total female goats was 59%. This is an increasing trend from the 31% reported earlier in the same study area, over two decades ago [12]. Muhammad *et* *al*. [18] reported a similar finding in goats in the North of Nigeria while lower rates were also reported by other researchers across Nigeria [10,13].

The pregnant does slaughtered were found to be higher in the second (51.9%) and third (29.9%) trimesters than in the first (18%) trimester. A similar finding was also observed by Mshelia *et al*. [10] and Adeyemi *et* *al*. [17]. This is surprising, given the fact that pregnancies in the second and third trimesters can be readily detected even if the first trimester may go unnoticed.

The general reason for the slaughter of pregnant does mainly in their 2^nd^ and 3^rd^ trimesters and their active reproductive age could be attributed to the high demand and preference for big-sized goats. Since pregnancy is associated with increased in size and body weight resulting from physiologic hypertrophy of the internal organs and tissues, lipogenesis, and body fluid retention [26], as mainly observed in the 2^nd^ and 3^rd^ trimester, the animals look robust and big. As a result of poverty, ignorance, and illiteracy of the livestock owners, they dispose of the supposedly big goats that are weighty and with shiny hair coat because they fetch a higher price, to meet their household needs which include payment of school fees, festivals, ceremonies, and agricultural activities [27].

The meat inspection act of 1968 prohibits the slaughter of pregnant animals, but there is a total lack of implementation of this act in Nigeria. The compensation paid to butchers by the government when such situations as pregnancy and disease conditions are encountered to discourage this practice of slaughtering and/or selling diseased animal to the consumers has long been stopped. Recently, most state governments in Nigeria have contracted out the meat inspections at abattoirs and slaughterhouses to private individuals who are only interested in generating revenue per head of animals slaughtered. Since no antemortem inspection is carried out in most Nigerian abattoirs, the proportion of fetal wastages observed in this study accounts for a considerable loss of animal protein and future national herd if similar occurrences from all abattoirs and slaughterhouses in all other states in the country are considered.

Moreso, the slaughter of pregnant animals is cruel and contrary to the principles of animal welfare. The quality of meat from pregnant animals has been reported to be poor as it contains high PH value, more tender, and watery and has poor eye appeal and sometimes abnormal smell due to high levels of pregnancy hormones in tissues [28]. Furthermore, animals in late pregnancy have lower mean initial yield and peak force shear values for *Longissimus dorsi* muscles than non-pregnant animals [28], hence the negative consequences of poor meat quality.

The economic implications of the slaughter of pregnant animals can be a reduction in the national herd size or negative growth rate of the livestock industry or a tremendous financial loss due to fetal wastage.

Our findings revealed that a total of 660 fetuses were wasted within the 3-month study duration (Table-3). This shows there is a reduction of 660 goats from the national herd from Nsukka slaughterhouse alone in just 3 months. However, when considering the financial loss due to fetal wastage, at the present cost of ₦6,000 ($16) and ₦8,000 ($20) for male and female kids, respectively, a total of ₦4,648,000 ($11,620) was lost in just one slaughterhouse in 3 months. Similar studies across Nigeria have reported varying degrees of fetal wastages and financial losses [10,13]. If considered nationwide, then certainly, the slaughtering of pregnant food animals has a serious economic and food security concerns to Nigerians and the Nigerian livestock economy.

## Conclusion

This study has shown a very high level of cruelty to animals in Nigeria as observed in the inhumane slaughter of pregnant goats for meat. It has revealed that the losses from the goat population are very high and increasing, as well as the quality of a higher percentage of the chevon sold to the consumers is poor. This indicates a serious threat to the Nigerian goat population and impacts negatively on the country’s economy, as the price of meat generally and chevon, in particular, will continue to increase. Hence, the protein malnutrition level in Nigeria will also continue to increase.

The total lack of implementation of the meat inspection act is grossly responsible for this menace. The government should take it very seriously and put all the necessary measures in place to implement this act as it will go a long way to minimizing and even eradicating the menace and cruelty of slaughtering pregnant animals especially goats for meat in the slaughterhouses and abattoirs in Nigeria.

## Authors’ Contributions

OJO, EEV, TEE, and OCO got the concept of the work, designed the study, did the data analysis and interpretation. CAA, EEV, COA, OJO, and SOO performed the lab analysis of the samples and CAA, COA, COO, TEE, and OJO drafted the manuscript. All authors read, corrected, and revised the manuscript.
